# Exploration of Genomic Regions Associated with Fusarium Head Blight Resistance in Wheat and Development and Validation of Kompetitive Allele-Specific Polymerase Chain Reaction Markers

**DOI:** 10.3390/ijms26073339

**Published:** 2025-04-03

**Authors:** Pengbo Song, Yueyue Li, Xin Wang, Xiaoxiao Wang, Aoyan Zhang, Zitan Wang, Wensha Zhao, Haoyang Li, Huiling Zhao, Kefeng Song, Yuanhang Xing, Xiaoran Guo, Xin Zhang, Shengjie Sun, Yi Feng, Daojie Sun

**Affiliations:** 1 College of Agronomy, Northwest A&F University, Yangling 712100, China; songpb@nwafu.edu.cn (P.S.); fengyiwheat@nwafu.edu.cn (Y.F.); 2 Xiangyang Academy of Agricultural Sciences, Xiangyang 441000, China

**Keywords:** wheat, FHB resistance, QTL, KASP marker

## Abstract

*Fusarium* head blight (FHB), caused by *Fusarium graminearum*, is a globally significant disease that severely impacts the yield and quality of wheat. Breeding resistant wheat varieties using resistance genes is the most cost-effective strategy for managing FHB, but few markers are available for marker-assisted selection (MAS) of resistance. In this study, we evaluated the resistance of a recombinant inbred line (RIL) population to FHB through single-floret inoculation in four field environments over two years. Combined with quantitative trait loci (QTL) detection through high-density genetic mapping based on wheat 50 K SNP arrays, we identified a total of 21 QTLs influencing FHB resistance. It is worth noting that *QFhba-5D.2-1* was detected in two field environments as well as in the multi-environment trial (MET) analysis, explaining phenotypic variation ranging from 1.98% to 18.55%. We also pinpointed thirteen resistance genes within the QTL intervals on chromosomes 4A, 5D, 6B, and 7A associated with FHB defense mechanisms. Furthermore, we developed two Kompetitive Allele-Specific PCR (KASP) markers for the *QFhba-5D.2-1* and *QFhba-7A* regions to validate their specificity within the RIL population. Subsequently, we validated the polymorphism of these two markers in 305 wheat germplasms and analyzed their effect on thousand kernel weight (TKW) and spike length (SL). These markers will accelerate the development of FHB-resistant wheat varieties through MAS, significantly reducing yield losses and strengthening food security.

## 1. Introduction

Fusarium head blight (FHB) is one of the most destructive diseases affecting wheat (*Triticum aestivum* L.) worldwide, leading to substantial reductions in grain yield and quality [1,2,3]. Statistical estimates suggest that FHB incurs approximately 22% of the total yield losses experienced during both the pre-harvest and post-harvest stages in wheat [4,5]. The shriveling, discoloration, and moldiness of wheat grains infected with FHB are caused by toxins produced by *Fusarium* species [6,7]. These mycotoxins disrupt the normal development and metabolism of the grains, resulting in lighter test weights and reduced yield [5,7]. Furthermore, toxins, such as deoxynivalenol (DON), produced in diseased seeds make them unsuitable as food or feed [8]. In recent years, the incidence of FHB has notably increased in China due to climatic influences, affecting many wheat-growing regions [9]. Breeding and planting resistant varieties have been recognized as the most economical and effective measures for managing FHB [10,11,12,13].

FHB resistance in wheat is a complex quantitative genetic trait influenced by multiple genes and is significantly affected by environmental factors [14,15]. It is influenced by spike morphology, spikelet compactness (SC), spike length (SL), the presence or absence of awns, and intrinsic traits, such as plant height (PH) and flowering date (FD) [5,15,16]. Additionally, the presence or absence of anther residues in the glumes after flowering and the degree of glume closure are closely related to FHB infection [10,17]. Environmental factors affecting wheat FHB include temperature, humidity, rainfall, ventilation, and crop planting density [5,18,19,20,21].

Exploring quantitative trait loci (QTL) affecting FHB resistance through association and linkage analyses is a critical step in developing new resistant germplass using molecular marker-assisted selection (MAS) [13]. Over 500 QTL affecting FHB resistance have been identified across all 21 wheat chromosomes [22,23,24,25]. However, fewer more than ten resistance loci have been formally designated, among which *Fhb1*, *Fhb2*, and *Qfhs.ifa-5A* were sourced from the Chinese wheat germplasm ‘Sumai-3’ [26], whereas ‘Wangshuibai’ contributed the resistance loci *Fhb4* [27] and *Fhb5* [10,28,29]. *Fhb3* originates from the wild relative *Leymus racemosus*, and it was introgressed onto the 7AS chromosome of wheat through traditional breeding and MAS [30,31]. *Fhb6* [32] and *Fhb7* [33] resistance genes were transferred from *Elymus tsukushiensis* and *Thinopyrum ponticum*, respectively, to the 1A and 7D chromosomes of wheat using techniques like chromosome engineering and MAS [10,31,34]. Furthermore, significant QTLs influencing FHB in wheat, including *Fhb8* [35], *Fhb9* [25], and *QFhb.hwwg-2DS* [36], have been reported in recent years. *Fhb1* exhibits the greatest Type II (resistance to fungal spread within the infected spikes) [37,38] resistance against FHB among all named QTLs and demonstrates stable resistance across multiple environments and different genetic backgrounds. Type I (resistance to initial infection) [11,15] and Type II resistance are governed by distinct genomic regions, and their combination enhances overall resistance to FHB. For example, *Fhb1* significantly reduces the incidence of FHB when it co-exists with the validated *Qfhs.ifa-5A* [37].

Relying on only one or a few resistance sources across large crop production areas increases the risk of resistance breakdown and subsequent disease epidemics. In contrast, molecular pyramiding of multiple resistance genes can significantly enhance disease resistance in crops. However, the number of QTL with major effects identified for molecular marker-assisted breeding remains limited [38]. Therefore, it is essential to identify QTL influencing FHB resistance in diverse wheat germplasms and develop Kompetitive Allele-Specific PCR (KASP) markers to enhance wheat breeding efforts for FHB resistance. This study utilized a high-density genetic map to conduct linkage analysis of traits influencing FHB across four environments over two years in a recombinant inbred line (RIL) population, which comprised 198 lines. The goal was to identify genomic regions associated with FHB and successfully develop and validate corresponding KASP markers. These findings hold the potential to provide critical insights for breeding wheat varieties with superior resistance to FHB.

## 2. Results

### 2.1. Phenotypic Analysis for FHB Resistance in the RIL Population

Phenotyping statistics and analysis of variance (ANOVA) for FHB across four distinct environments over a two-year period indicated that AS and PSS demonstrated a continuous distribution and transgressive segregation within the RIL population, consistent with principles observed in quantitative genetics research (Figure S1). Comparison to XN1376, XY81 exhibited higher FHB AS and higher PSS. In four environments, the average phenotypic coefficient of variation (CV) for AS was 0.54, with a genetic variance of 0.45 and a heritability of 0.70, suggesting a relatively stable phenotype with a strong genetic influence. In contrast, the CV for PSS was 0.60, with a genetic variance of 13.27 and a heritability of 0.62, indicating greater phenotypic and genetic variation, with the phenotype influenced by both genetic and environmental factors (Table 1). We analyzed the phenotypic correlations between AS and PSS across four different environments and found a statistically significant correlation between AS and PSS for FHB in all environments. The phenotypic correlation coefficients for AS and PSS were higher within the same year, ranging from 0.66 to 0.92. In contrast, across different years under the same sowing period, the correlation coefficients for AS ranged from 0.42 to 0.68, while those for PSS ranged from 0.84 to 0.85 (Figure S2).

### 2.2. QTL Analysis

**Figure 1:**
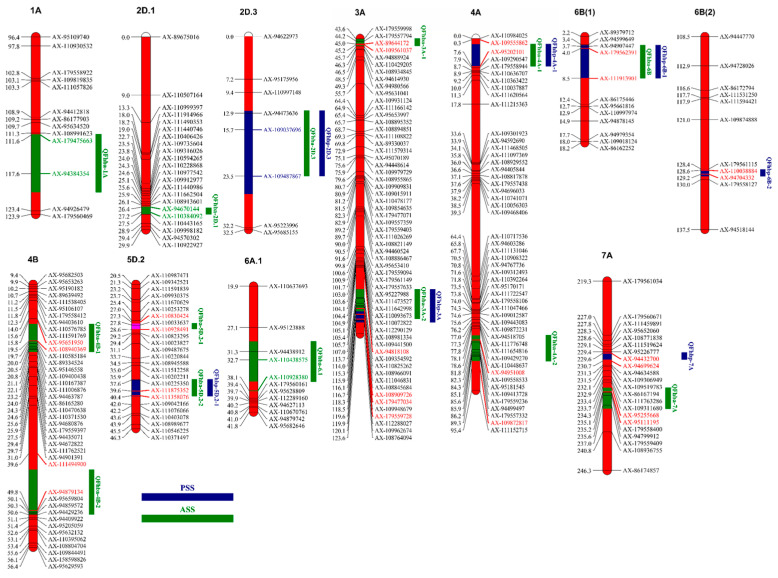
Distribution of QTL on chromosomes identified through linkage analysis.

QTL analyses of phenotypic data from four environments over two years and BLUP values detected a total of 21 QTLs affecting FHB. Fourteen AS-associated QTLs were identified on chromosomes 1A, 2D (2), 3A (2), 4A (2), 4B (2), 5D (2), 6A, 6B, and 7A. Additionally, seven PSS-associated QTLs were detected on chromosomes 2D, 3A, 4A, 5D, 6B (2), and 7A (Table 2; Figure 1; Table S1). In addition, multi-environment trait QTL analyses were conducted, revealing that 13 QTLs overlapped with the QTL positions identified in a single environment (Table S2).

Among the 14 QTLs identified for AS, *QFhba-1A*, *QFhba-2D.1*, *QFhba-2D.3*, *QFhba-3A-1*, *QFhba-3A-2*, *QFhba-4A-1*, *QFhba-4B-1*, *QFhba-4B-2*, and *QFhba-6A.1* were detected only in single environments (without MET), with all of their resistance alleles derived from XY81. The range of phenotypic variance explained was 0.98% to 7.20%. *QFhba-3A-2*, *QFhba-4A-2*, *QFhba-4B-2*, and *QFhba-6A.1* were located in close proximity to or overlapped with QTL positions detected through MET across different environments. The resistance alleles of *QFhba-4A-2*, *QFhba-5D.2-1*, *QFhba-5D.2-2*, *QFhba-6B*, and *QFhba-7A*, which were detected in at least two environments, were derived from XN1376. Among them, *QFhba-5D.2-1* was identified as a major-effect QTL with a phenotypic variance explanation rate exceeding 10%. The position of *QFhba-4A-2* on the genetic map was 82–87 cM, corresponding to 543.8–621.8 Mb in the reference genome. In contrast, *QFhba-5D.2-2* spanned only 0.5 Mb, which was significantly smaller than the physical interval of *QFhba-5D.2-1* (469.5–573.8 Mb). Additionally, the physical intervals occupied by both *QFhba-6B* and *QFhba-7A* were less than 20 Mb.

Among the seven QTLs identified for PSS, *QFhbp-2D.3*, *QFhbp-5D.2-1*, *QFhbp-6B-1*, and QFhbp-7A were consistently detected in at least two environments (including BLUP). Notably, of these four QTLs, only *QFhbp-2D.3* exhibited a resistance allele derived from XY81. *QFhbp-3A*, *QFhbp-4A*, and *QFhbp-6B-2* were minor QTLs detected in a single environment. The QTLs detected for AS on chromosomes 2D, 3A, 4A, 5D, 6B, and 7A are located near or overlap with the QTLs detected for PSS on the same chromosomes.

### 2.3. Effect Analysis of QFhba-5D.2-1 with QFhba-7A and QFhbp-7A

**Figure 2:**
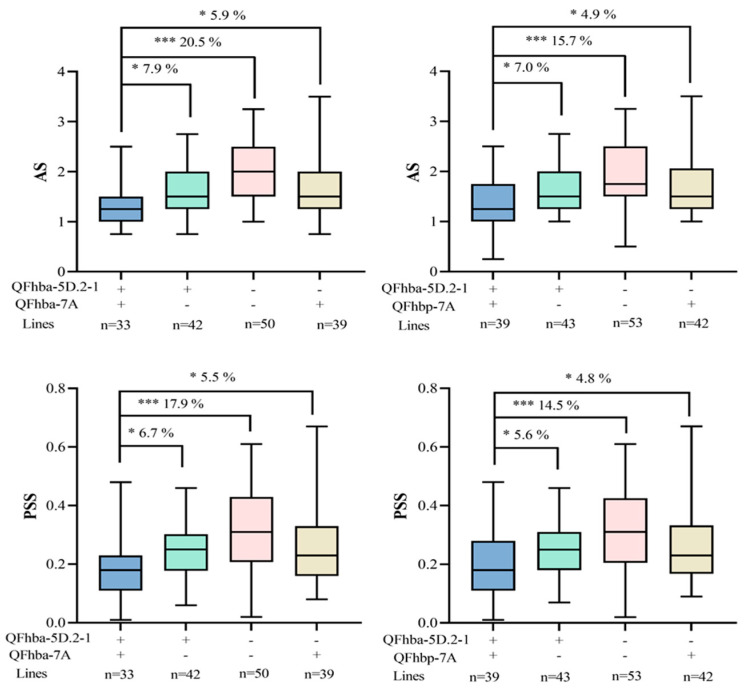
The polymeric additive effects of *QFhba-5D.2-1* with *QFhba-7A* and *QFhbp-7A* were analyzed in the RIL population. +: resistance allele of the corresponding flanking marker derived from XN1376; −: susceptible allele of the corresponding flanking marker derived from XY81; *** *p* < 0.0001, * *p* < 0.05; AS, average severity; PSS, percentage of symptomatic spikelets.

The stability of the QTLs identified in this study was further confirmed through QTL-environment interaction analysis, where all QTLs detected in individual environments were also detected through ‘MET’ (Table S2). QTL effect analysis, using flanking markers for *QFhba-5D.2-1*, *QFhba-7A*, and *QFhbp-7A*, combined with phenotypic means from multiple environments, indicated that lines carrying the *QFhba-5D.2-1* resistance allele exhibited a 4.2% increase in AS resistance and a 5.0% increase in PSS resistance. Lines carrying the *QFhba-7A* resistance allele showed a 6.4% increase in AS resistance and a 4.7% increase in PSS resistance. Similarly, lines carrying the *QFhbp-7A* resistance allele displayed a 5.1% increase in AS resistance and a 3.7% increase in PSS resistance (Figure S3). Notably, we analyzed the additive effect of combining the *QFhba-5D.2-1* locus with either the *QFhba-7A* or the *QFhbp-7A* resistance locus. The results showed that compared to lines carrying the susceptibility alleles of both *QFhba-5D.2-1* and *QFhba-7A*, lines carrying the resistance alleles of both loci exhibited a 20.5% increase in AS resistance and a 17.9% increase in PSS resistance. Additionally, lines carrying the resistance alleles of *QFhba-5D.2-1* and *QFhbp-7A* showed a 15.7% increase in AS resistance and a 14.5% increase in PSS resistance compared to lines with the susceptibility alleles of both loci (Figure 2).

### 2.4. Development and Validation of KASP Marker

**Figure 3:**
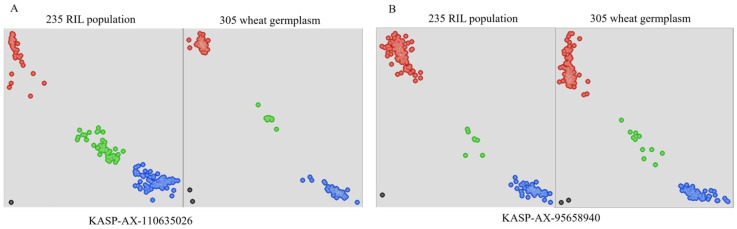
Genotyping of two KASP markers in the RIL population and the 305 wheat germplasm accessions. The red point in (**A**) represents genotype C/C, the blue point represents genotype T/T, and the green points represent heterozygosity. In (**B**), the red point represents genotype A/A, the blue point represents genotype G/G, and the green point represents heterozygosity.

**Figure 4:**
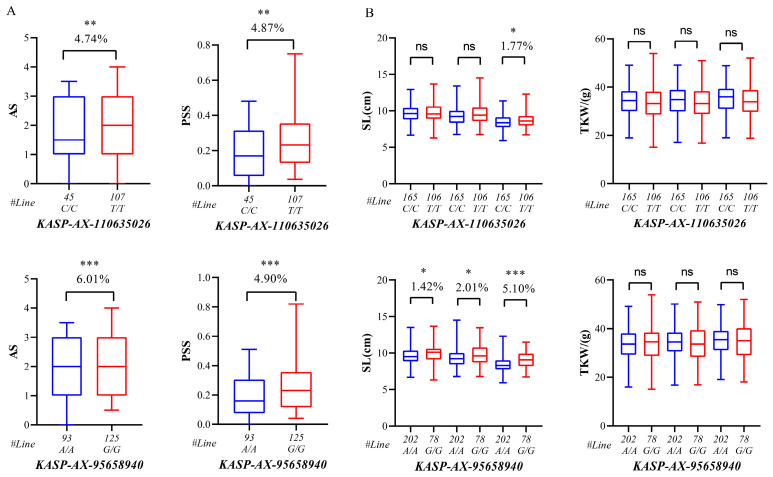
Box and line diagrams (**A**) represent the comparison of phenotypic differences (AS, PSS) between different pure genotypes of the two KASP markers in recombinant inbred lines (RILs), and box and line diagrams (**B**) represent the effect of typing results of the two markers in 305 wheat germplasm materials on spike length (SL) and thousand kernel weights (TKW) in different environments. *** *p* < 0.0001, ** *p* < 0.001, * *p* < 0.05, ns, no significant difference between groups.

Based on the SNP markers with polymorphisms between both parents within the QFhba-5D.2-1 and QFhba-7A regions, we converted them into KASP markers. We screened more than 25 KASP primer pairs, but only two were successfully validated for typing in 235 RIL materials: *KASP-AX-110635026* and *KASP-AX-95658940* (Table S3). These two developed KASP markers were validated in 235 RILs, and the phenotypes were analyzed using the SNP typing results in combination with AS and PSS (Figure 3). We discovered that the phenotypes associated with different genotypes corresponding to *KASP-AX-110635026* and *KASP-AX-95658940* exhibited significant differences. Additionally, further analysis using these two markers across 305 wheat germplasm resources confirmed their reliability, demonstrating that they have no negative impact on TKW across various genetic backgrounds. However, *KASP-AX-110635026* had a significantly lower effect on SL compared to *KASP-AX-95658940*. The G/G genotype of *KASP-AX-95658940* was found to enhance SL while reducing resistance to FHB, indicating a potential antagonistic relationship between these traits. Therefore, the *KASP-AX-110635026* marker was considered more suitable for molecular marker-assisted selection breeding aimed at FHB resistance, as it did not adversely affect TKW or SL (Figure 3 and Figure 4).

### 2.5. Analysis of Genes Within the Genomic Regions of 4A, 5D, 6B, and 7A QTL Regions

In this study, we aimed to predict candidate genes within the 4A, 5D, 6B, and 7A QTL regions by analyzing the expression levels, expression patterns, and functional annotations of high-confidence genes within these regions. The results revealed that the 4A candidate region contained 30 candidate genes, the two QTL candidate regions on 5D contained 8 and 657 candidate genes, respectively, the 6B candidate region included 168 candidate genes, and the 7A candidate region contained candidate genes (Table S4).

## 3. Discussion

In this study, to determine the novelty of the detected QTLs, we collected over 2000 QTLs reported to influence FHB resistance from the literature and compared the physical positions of their flanking markers on the reference genome (Tables S1 and S5).

### 3.1. Comparison with Previous Studies

The resistance allele detected in a single environment originated from *QFhb-1A* of XY81, located within the 20.0–24.3 Mb interval on chromosome 1A of the reference genome. We compared the previously reported FHB resistance QTL in this region and found that the flanking markers, *BS00039749_51* and *RAC875_c64603_663*, are located within this QTL interval [39,40].

We identified *QFhba-2D.1* within the 233.4–349.6 Mb interval on chromosome 2D, overlapping with previously reported FHB-influencing regions [40,41,42]. *QFhba-2D.3* and *QFhbp-2D.3* were co-localized within the 18–20 cM interval, partially overlapping with the location of the marker (*IWB28458*) *EXCALIBUR_C6681_580*, which had been previously reported to affect FHB Type II resistance in the same physical region [43]. We hypothesize that this region may harbor genes crucial for enhancing FHB resistance, although the candidate genes remain unconfirmed.

*QFhba-3A-1* was identified on chromosome 3A within the 721.6–722.3 Mb interval, which co-localizes with the Type II FHB resistance-associated marker *IWB43218*, previously reported in the same region [44]. *QFhba-3A-2* and QFhbp-3A were co-localized within the 657.9–683.3 Mb interval on chromosome 3A. QTLs associated with Type II and Type III FHB resistance were reported in the same region [45,46,47,48,49]. Therefore, we hypothesize that it may be a QTL influenced by the same gene.

*QFhba-4A-1* and QFhbp-4A were co-localized at the 7 cM position on chromosome 4A of the genetic map. Markers affecting FHB resistance (including results from the mate-QTL study) were reported within the same physical interval (712.9–717.8 Mb) [40,48,49,50,51,52]. The physical interval of *QFhba-4A-2* overlapped with previously reported markers associated with Type II and Type III FHB resistance [2,22,47,48,53,54,55,56,57,58,59]. These findings suggest that these QTL may be influenced by the same gene.

*QFhba-4B-1* was identified as a micro-effective QTL in the 10.7–12.2 Mb region of chromosome 4B, overlapping with regions where markers influencing FHB resistance have been identified in previous studies [60]. This variation could be attributed to differences in the research materials and population size, leading to varying effect sizes. The *QFhba-4B-2* on chromosome 4B, spanning 25.8–63.2 Mb, included the *Rht-B1* gene and was associated with more than 25 reported QTLs influencing FHB resistance [2,17,39,48,57,59,61,62,63,64,65,66,67,68,69,70].

*QFhba-5D.2-1*, identified in multiple environments, accounts for 1.98–18.55% of the phenotypic variation. Approximately 40 QTLs affecting resistance to Type II and Type III FHB resistance were reported within the adjacent physical interval [13,25,40,43,44,48,51,55,63,71,72,73,74,75,76,77]; however, no KASP markers for FHB resistance detection were reported in this interval. The development of KASP markers for breeding in this major effect region is essential. *QFhba-5D.2-2* and *QFhbp-5D.2-1* were micro-effective QTLs, both localized within the 458.1–458.6 Mb region of chromosome 5D. Their physical locations fall within the intervals of QTLs for FHB resistance reported in previous studies, and *Vrn-D1* is also located within this region [22,61,76,78,79]. Therefore, we hypothesize that the variation may be influenced by this gene.

The micro-effective resistance locus *QFhba-6A.1*, provided by XY81, spans the 25.8–63.2 Mb region. This interval overlaps with markers like *IWB22389*-*IWA621*, which were known to influence both Type II and Type III FHB resistance [38,48,80,81].

*QFhba-6B* and *QFhbp-6B-1* were co-localized at 7–8 cM on the genetic map, with their flanking markers spanning a physical interval of 17.2 Mb. This region encompasses resistance QTLs that were documented to influence FHB in previous studies [2,39,40,58]. *QFhbp-6B-2* was a micro-effect QTL localized within a physical interval ranging from 8.0 to 11.3 Mb, which overlaps with regions identified in previous studies as influencing the incidence (INC), severity (SEV), and incidence index (IND) of FHB [58,59].

*QFhba-7A* and *QFhbp-7A* were co-located within an interval spanning 230 to 235 cM and exhibited stability across various environmental conditions. Together, they accounted for 0.97% to 6.85% of the phenotypic variation, with additive effects originating from XY81. In neighboring physical regions of this interval, studies reported QTLs associated with FHB resistance [13,46,50,55,58,60,70,82,83,84]. In this region, no KASP markers for the detection of FHB resistance were reported. These findings suggest that the molecular markers associated with this QTL are suitable for development as molecular markers for marker-assisted selection.

MET analyses of AS and PSS revealed that the QTL positions of AS and PSS almost completely overlapped after removing environmental factors. In addition, the number of QTL detected in the multi-environment analysis was significantly higher than that found in the single-environment study. The increase in the number of QTL may be due to environmental factors or genotype–environment (G × E) interactions, which may prevent certain QTL from being detected in a single environment. Therefore, MET analyses reveal more potential QTL and allow for a more accurate assessment of their stability across environments. QTL that can be detected in both single-environment and cross-environment assays can be considered multi-environment stable QTL, exhibiting a broad range of adaptations and resistances. These stable QTL are valuable in molecular breeding. Molecular markers developed based on these QTL can help in selecting varieties carrying favorable genes, enhancing disease resistance and adaptability, and ultimately speeding up the breeding process and improving crop yields.

QTL effect analyses of *QFhba-5D.2-1*, *QFhba-7A*, and *QFhbp-7A* demonstrated that combining their resistance alleles significantly enhances FHB resistance in wheat lines. Additionally, the developed KASP markers were successfully validated across diverse genetic backgrounds, offering a reliable tool for future breeding programs. These findings provide a solid foundation for selecting and improving FHB-resistant wheat varieties. In addition, the developed KASP markers have been successfully validated across different genetic backgrounds, providing a reliable tool for future wheat breeding programs. However, trade-offs may exist between FHB resistance and key traits, such as thousand kernel weight (TKW) and spike length (SL), in wheat breeding. Specifically, the G/G genotype of the *KASP-AX-95658940* marker can enhance SL but may reduce FHB resistance. In contrast, the *KASP-AX-1110635026* marker improves FHB resistance without negatively affecting TKW or SL, demonstrating its superior breeding potential. Therefore, breeding strategies should prioritize the *KASP-AX-1110635026* marker to strike a balance between resistance and yield traits. Meanwhile, genomic selection technology can optimize the improvement of multiple traits, providing a solid foundation for the development of FHB-tolerant wheat varieties. By combining genomic selection with KASP markers, breeders can more precisely select for target traits, thereby accelerating the wheat breeding process and enhancing both resistance and yield advantages.

### 3.2. Candidate Genes Involved in Plant Defense Responses to Pathogens

Functional screening of high-confidence genes within the QTL candidate regions identified genes encoding NBS-LRR disease resistance proteins and NBS-LRR disease resistance protein-like proteins in segments on chromosomes 4A, 5D, 6B, and 7A (Table S6). Previous studies have reported these genes to play a role in the defense mechanism against FHB [13,85]. The identification of candidate genes in this study provides valuable insights into the mechanisms underlying FHB resistance in wheat, laying the groundwork for future functional annotation and phenotypic analysis. Moreover, this discovery facilitates the exploration of these genes’ roles in disease resistance and the validation of their functions in subsequent research.

## 4. Materials and Methods

### 4.1. Plant Materials and Trial Environments

In this study, we utilized 198 lines from the RIL population derived from the cross between ‘Xinong 1376’ (XN 1376) and ‘Xiaoyan 81’ (XY 81) for QTL mapping. XN1376 is a semi-vernal variety with resistance to FHB recognized for its early maturity (which helps it avoid the peak disease incidence period) as well as its broad adaptability and superior agronomic traits. In contrast, XY81, which demonstrates moderate resistance to FHB, is distinguished by strong adaptability, efficient nutrient utilization, lodging resistance, and timely maturation. The experimental materials included 198 RILs for QTL mapping, 235 lines and parental varieties for KASP validation, and 305 wheat germplasm accessions (Table S7). All materials were planted at the Agricultural One-Stop Experimental Base of Northwest A&F University in Yangling (34°18′ N, 108°04′ E) during the 2020–2021 (E1/E2) and 2021–2022 (E3/E4) cropping seasons. In addition, these 305 wheat germplasm resources were also planted during the 2022–2023 (E5) cropping season. The soil is classified as calcaric regosol [86]. After wheat harvest, the land remains fallow until the next wheat planting season, during which subsoiling is conducted to improve soil structure and enhance water infiltration. The planting periods consisted of normal and late sowing, with the late-sown lines planted one month later than the normal sowing period. Notably, significant differences in environmental conditions were observed between the two sowing periods. A randomized complete block design with two replications for each sowing period each year was employed. Each variety (line) was planted using a two-row single-seed sowing method, with a sowing density of 30 seeds per row, a row length of 2 m, and row spacing of 23 cm. The experimental materials were planted and managed following the methods reported in previous studies [87].

### 4.2. Field Inoculation

The four highly pathogenic strains (F0980, F1312, F0609, and F0301) used in the experiment were identical to those employed in the national wheat regional trial (kindly provided by Dr. Guihong Yin, Henan Agricultural University). Inoculation was performed using the single-floret inoculation method; the spore solution of the four pre-cultured strains was mixed and diluted to a concentration of 1 × 10^5^ spores per mL. Subsequently, 10 μL of the suspension was injected into the outermost flower of the fourth spikelet located in the upper-middle portion of the wheat spike during the flowering stage. Following inoculation, the spikes were sprayed with water and covered with plastic bags to maintain moisture for 72 h. After removing the bags, the spikes were periodically sprayed with water to sustain humidity. A total of ten spikes were inoculated on 3–5 plants from each line or variety, and the experiment was replicated twice.

### 4.3. Phenotypic Data Collection

Twenty-one days after inoculation, the number of diseased spikes and the number of spikelets per spike were assessed. The mean percentage of symptomatic spikelets (PSS) and mean disease severity were then calculated. The percentage of symptomatic spikelets was calculated as follows: PSS = (number of diseased spikelets/total number of spikelets) × 100%. We modified the evaluation method proposed by Sari et al. [88] to assess the average severity (AS) of FHB in each plant. Severity was visually evaluated based on the grade of infected spikes (including the susceptibility of the spike rachis), with grades ranging from 0 to 4 indicating levels from resistance to spread (or disease escape) to high susceptibility. At maturity, 10 healthy main spikes from each line in the set of 305 wheat germplasm accessions were randomly selected, and their lengths were measured using a straightedge. The length was measured from the base of the first resultant spikelet to the tip (excluding the awn) and recorded in centimeters. The mean spike length (SL) was then calculated. Between 15 to 20 spikes were collected, threshed, and dried in the sun. Subsequently, the thousand kernel weight (TKW) was measured using the Wanshen SC-G Seed Examiner (Hangzhou Wanshen Inspection Technology Co. Ltd., Hangzhou, China).

### 4.4. Phenotypic Analysis and QTL Analysis

Descriptive statistics were conducted for the phenotypic data using IBM SPSS Statistics 20. In addition, the lme4 package in R 4.3.2 was used to compute the Best Linear Unbiased Prediction (BLUP), estimate variance components, and calculate heritability (h^2^) using the following formula h2 = Vg/(Vg + Vge/l + (Ve/r l)), where Vg represents genetic variance, Vge represents genotype–environment interaction variance, Ve represents residual variance, l indicates the number of environments, and r represents the repeat factor. The analysis also included the estimation of genetic coefficients of variation (CV) and the calculation of correlations between traits across different environments [87].

The genetic map used in this study was constructed using the 50 K SNP chip, as reported in previous studies [89]. It encompasses a total length of 3605.53 centimorgans (cM) and consists of 28 linkage groups, with an average marker spacing of 1.34 cM. This map covers all 21 chromosomes of wheat. The ‘BIP’ module in ICI-mapping 4.2 software employs the inclusive composite interval mapping with the additive (ICIM-ADD) method to identify QTL in single environments, while the ‘MET’ module is used to detect QTL across multiple environments. The ICIM-ADD method was executed with a step size of 0.1 cM and a LOD score threshold of 2.5 for QTL detection. We adhered to the standard QTL naming convention [90]: ‘Q’ + ‘trait abbreviation’ + hyphen (‘-’) + ‘chromosome designation.’ When a chromosome contains multiple linkage groups, they are separated by a dot (‘.’), and when more than one QTL is detected within the same linkage group, they are numbered sequentially (e.g., -1, -2). For example, ‘QFhba-5D.2-2’ represents the second QTL identified for the ‘Fhba’ trait on the second linkage group of chromosomes 5D. We defined QTLs with a phenotypic variance explanation greater than 10% as major-effect QTL. The novelty of the identified QTL in this study was evaluated by comparing their physical locations with those reported in previous studies. The locations of QTL on chromosomes were visualized using Mapchart 2.3.2 (https://www.wur.nl/en/show/mapchart.htm, accessed on 17 February 2025).

### 4.5. Candidate Gene Prediction

The QTL regions identified through linkage analysis across multiple environments were mapped to reference genome version 1.0, which is available on the WheatOmics (Wheat Gene Expression Database) website (http://202.194.139.32/expression/wheat.html, accessed on 17 February 2025) [91]. High-confidence genes influencing FHB were identified by screening the Wheat EXP database (http://www.wheat-expression.com/, accessed on 17 February 2025) [92] and the Wheat eFP database (http://bar.utoronto.ca/efp_wheat/cgi-bin/efpWeb.cgi, accessed on 17 February 2025) [93], specifically targeting genes expressed in tissues associated with FHB susceptibility (TPM > 0.5).

### 4.6. KASP Marker Development

In this study, we mapped the physical locations of QTL flanking markers, initially identified with the 50 K chip, to the corresponding physical intervals on the biparental 660 K chip. Additionally, selected SNPs within the QTL region that exhibited polymorphism between the two parental lines were converted into KASP markers using the Poly Marker website (http://www.polymarker.info/, accessed on 17 February 2025). After the successful conversion of SNPs into KASP markers, the addition of FAM (5′ GAAGGTGACCAAGTTCATGCT 3′) and HEX (5′ GAAGGTCGGAGTCAACGGATT 3′) was required to ensure reliable allele identification. The primer concentrations, PCR amplification conditions, and procedures were followed according to methods described in previous studies [94,95].

## 5. Conclusions

In this study, we have identified 21 QTL regions for FHB resistance traits, all of which may have utility for further breeding applications. Most importantly, the six QTL regions on chromosomes 2D, 4A, 5D (2), 6B, and 7A were the most consistent among all of the detected marker–trait associations based on the criteria discussed above, and the 13 genes that may affect FHB resistance were located within these QTL intervals. The QTL and physical location data collected for FHB in wheat also serve as valuable references for future research in this area. Moreover, breeder-friendly KASP assays were developed and validated for *QFhba-5D.2-1* and *QFhba-7A* (*QFhbp-7A*). The discovery of these QTLs and candidate genes significantly enhances the genetic diversity underlying FHB resistance, which is crucial for addressing potential FHB epidemics in future production. Additionally, the developed KASP markers can be utilized for the pyramiding of FHB resistance genes and marker-assisted selection breeding. By employing genomic breeding strategies, quantitative traits from various resistance sources can be efficiently combined, thereby improving FHB resistance across different varieties, accelerating the stacking of multiple resistance genes, and advancing the development and improvement of new cultivars.

## Figures and Tables

**Table 1:** Phenotypic variation, heritability, and coefficient of variation of FHB in parents and RIL populations.

FHB Trait	Env^a^	XN1376	XY81	RILs					
				Min–Max	Mean ± SD	Vp	CV	Vg	*h* ^2^
AS	E1	2.00	3.00	0–4.00	1.88 ± 0.99	0.86	0.54	0.45	0.70
	E2	2.00	3.00	0–4.00	2.09 ± 1.00	0.98			
	E3	2.00	3.00	0–4.00	1.39 ± 0.77	0.55			
	E4	1.00	2.00	0–4.00	1.43 ± 0.91	0.68			
PSS (%)	E1	28.00	47.00	2.38–100.00	34.80 ± 25.67	655.98	0.60	13.27	0.62
	E2	25.30	40.70	3.45–100.00	39.77 ± 24.53	601.48			
	E3	15.25	23.00	3.35–78.72	27.08 ± 15.13	226.51			
	E4	10.00	17.45	4.29–62.52	26.12 ± 13.14	172.58			

Env^a^ 2020–2021(E1/E2), 2021–2022(E3/E4); RILs represent lines from the RIL population; Vp, phenotypic variance; Vg, genetic variance; *h*^2^, broad-sense heritability; CV, phenotypic coefficients of variation in multiple environments; AS, average severity; PSS, percentage of symptomatic spikelets.

**Table 2:** The distribution of QTLs detected in the RIL population on chromosomes.

QTL	Chro	Env	Position (cM)	Marker Interval	LOD Range	PVE Range (%)	Add Range	Confidence Interval
QFhba-1A	1A	E1	116	AX-179475663~AX-94384354	3.03	3.71	0.77	111.5~120.5
QFhba-2D.1	2D.1	E4	27	AX-94670144~AX-110384092	2.95	7.46	0.18	26.5~27.5
QFhba-2D.3	2D.3	BLUP	18	AX-109037696~AX-109487867	3.91	5.10	0.04	12.5~23.5
QFhba-3A-1	3A	E1	45	AX-89644172~AX-109561037	3.15	3.51	0.75	43.5~45.5
QFhba-3A-2	3A	E3/MET	113~118	AX-94818108~AX-179477034	2.51~2.73	0.98~6.20	0.07~0.17	111.5~119.5
QFhba-4A-1	4A	E3/MET	7	AX-109555862~AX-95202101	2.87~5.84	1.95~7.20	0.04~0.11	1.5~7.5
QFhba-4A-2	4A	E1/E2/MET	82~87	AX-94951008~AX-109872817	5.54~6.58	3.01~7.20	−0.01~−1.03	80.5~87.5
QFhba-4B-1	4B	E3/MET	19	AX-95651950~AX-108940369	3.43~4.14	1.56~6.91	0.10~0.21	16.5~21.5
QFhba-4B-2	4B	E1	49	AX-111494900~AX-94879134	5.45	6.49	1.03	42.5~50.5
QFhba-5D.2-1	5D.2	E3/E4/BLUP/MET	28	AX-110830424~AX-110928491	3.83~6.30	1.98~18.55	−0.10~−0.64	27.5~28.5
QFhba-5D.2-2	5D.2	E3/E4/BLUP	40	AX-111875352~AX-111358076	4.10~5.59	4.85~6.54	−0.04	37.5~40.5
QFhba-6A.1	6A.1	E2/MET	33~34	AX-110438575~AX-110928380	2.95~3.28	1.45~6.07	0.08~0.27	29.5~36.5
QFhba-6B	6B	E2/E3/E4/BLUP/MET	7~8	AX-179562391~AX-111913901	3.56~5.95	2.52~9.86	−0.03~−0.34	3.5~8.5
QFhba-7A	7A	E1/E3/BLUP/MET	235	AX-95255668~AX-95111195	4.00~5.61	1.97~6.48	−0.08~−1.04	234.5~237.5
QFhbp-2D.3	2D.3	E4/BLUP/MET	18~20	AX-109037696~AX-109487867	3.91~7.25	2.19~5.33	0.02~0.17	12.5~26.5
QFhbp-3A	3A	E3/MET	118~119	AX-108909726~AX-179559728	2.76~4.03	1.33~6.76	0.02~0.04	117.5~120.5
QFhbp-4A-1	4A	E3/MET	7	AX-109555862~AX-95202101	3.33~4.35	1.08~7.44	0.02~0.06	1.5~7.5
QFhbp-5D.2-1	5D.2	E3/E4/BLUP	40	AX-111875352~AX-111358076	4.02~5.59	5.37~5.92	−0.04	37.5~40.5
QFhbp-6B-1	6B	E2/E4/MET	7~8	AX-179562391~AX-111913901	3.56~6.58	3.32~9.08	−0.02~−0.08	3.5~8.5
QFhbp-6B-2	6B	E3	129	AX-110038884~AX-94704332	3.96	7.23	0.04	128.5~129.5
QFhbp-7A	7A	E1/E3/MET	230	AX-94432700~AX-94699624	3.63~4.21	0.97~6.85	−0.04	229.5~230.5

Chro, chromosome; LOD, logarithm of odds; PVE, phenotype variance explained; Add range, additive effect range of same interval, QTL; positive values: alleles from XN1376 are increasing the trait scores; negative values: alleles from XY81 are increasing the scores; BLUP, Best Linear Unbiased Prediction; MET, assessment of QTL after multi-environmental effects.

## Data Availability

The data supporting the findings of this study are available from the corresponding author (Dr Daojie Sun) upon request.

## Supplementary Materials

The following supporting information can be downloaded at https://www.mdpi.com/article/10.3390/ijms26073339/s1.

## Abbreviations

The following abbreviations are used in this manuscript:

AS	average severity
BLUP	Best Linear Unbiased Prediction
CV	coefficients of variation
FD	flowering date
FHB	Fusarium head blight
ICIM-ADD	inclusive composite interval mapping with the additive
KASP	Kompetitive Allele-Specific PCR
MAS	marker-assisted selection
MET	multi-environment trial
PH	plant height
PSS	percentage of symptomatic spikelets
QTL	quantitative trait loci
RIL	recombinant inbred line
SC	spikelet compactness
SL	spike length
TKW	thousand kernel weight
XN 1376	Xinong 1376
XY 81	Xiaoyan 81
AS	average severity
