# Supplementary material for: Exploration of Genomic Regions Associated with Fusarium Head Blight Resistance in Wheat and Development and Validation of Kompetitive Allele-Specific Polymerase Chain Reaction Markers
Source: Int J Mol Sci. 2025 Apr 3;26(7):3339. doi: 10.3390/ijms26073339 (PMC11989977; doi:10.3390/ijms26073339)
Supplement: Supplementary file 1 [file ijms-26-03339-s001.zip › supplementary figures.pdf]

Supplementary Figure. S1 Histograms of the phenotypic distribution of FHB-related traits in four environments over two years.

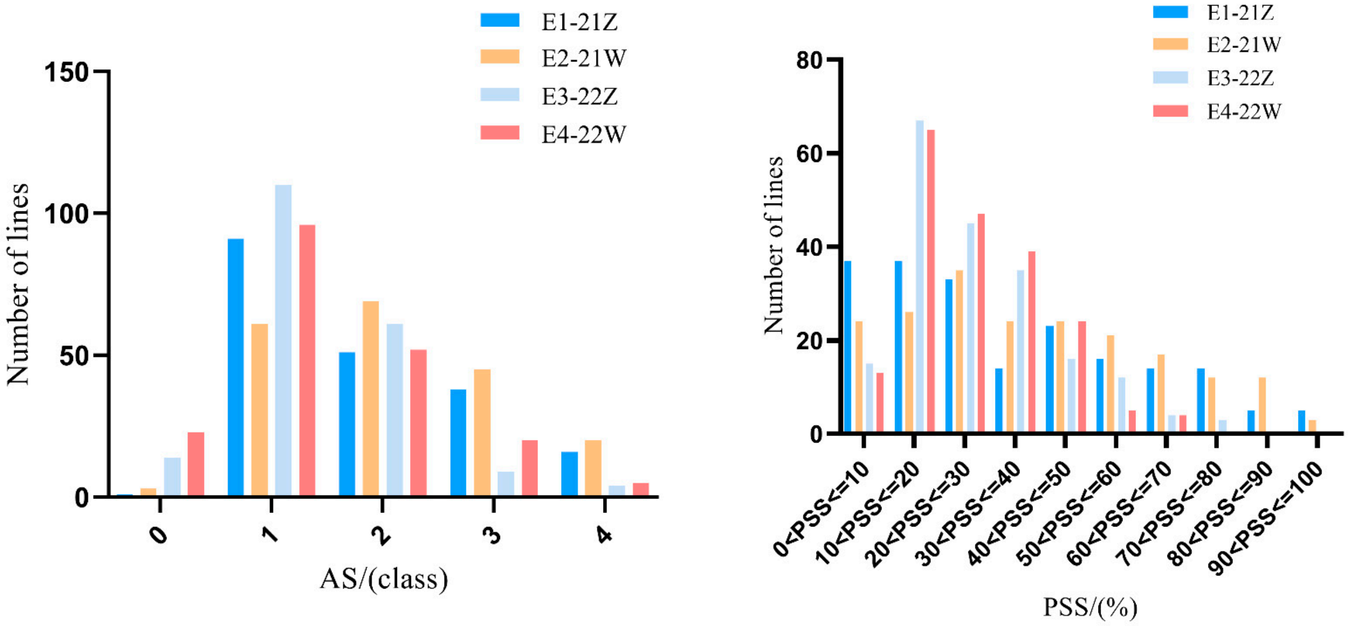

Figure S1. The horizontal coordinate of histogram A represents the average severity (AS), and the vertical coordinate is the number of lines; the horizontal coordinate of histogram B represents the percentage of symptomatic spikelets (PSS), and the vertical coordinate is the number of lines. In the notation 21Z,21W, 'Z' represents normal sowing, while 'W' indicates one month of late sowing.

Supplementary Figure. S2 Correlation coefficients of Fusarium head blight traits in the XN1376 × XY81 population across four different environments.

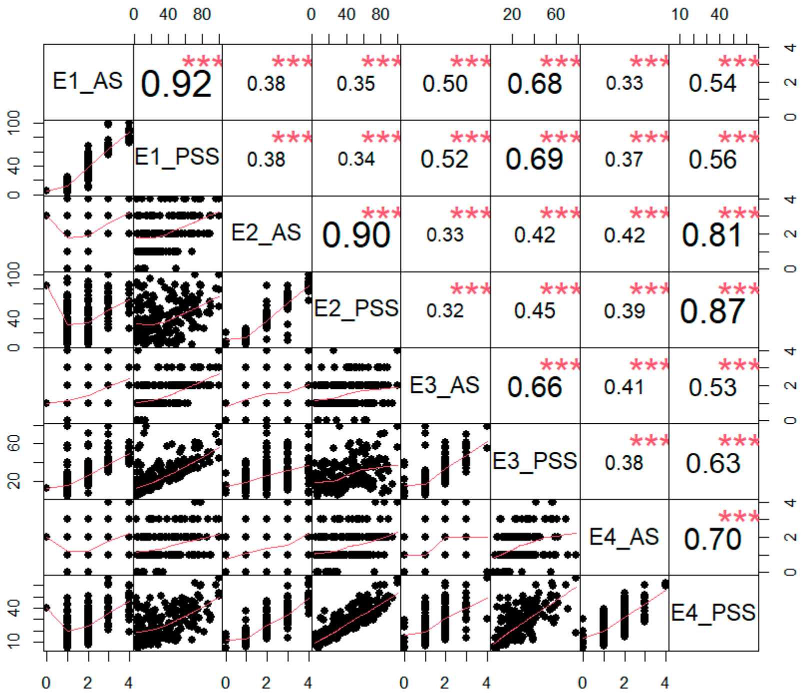

Figure S2. The X–Y scatter plots in the lower left panels show the correlations between traits, while the corresponding Pearson's correlation coefficients and *p* values of multiple comparison tests are located in the upper right panels. $^*p < 0.05$, $^{**}p < 0.01$, $^{***}p < 0.001$

Supplementary Figure. S3 QTL effect analysis of *QFhba-5D.2-1*, *QFhba-7A*, and *QFhbp-7A*

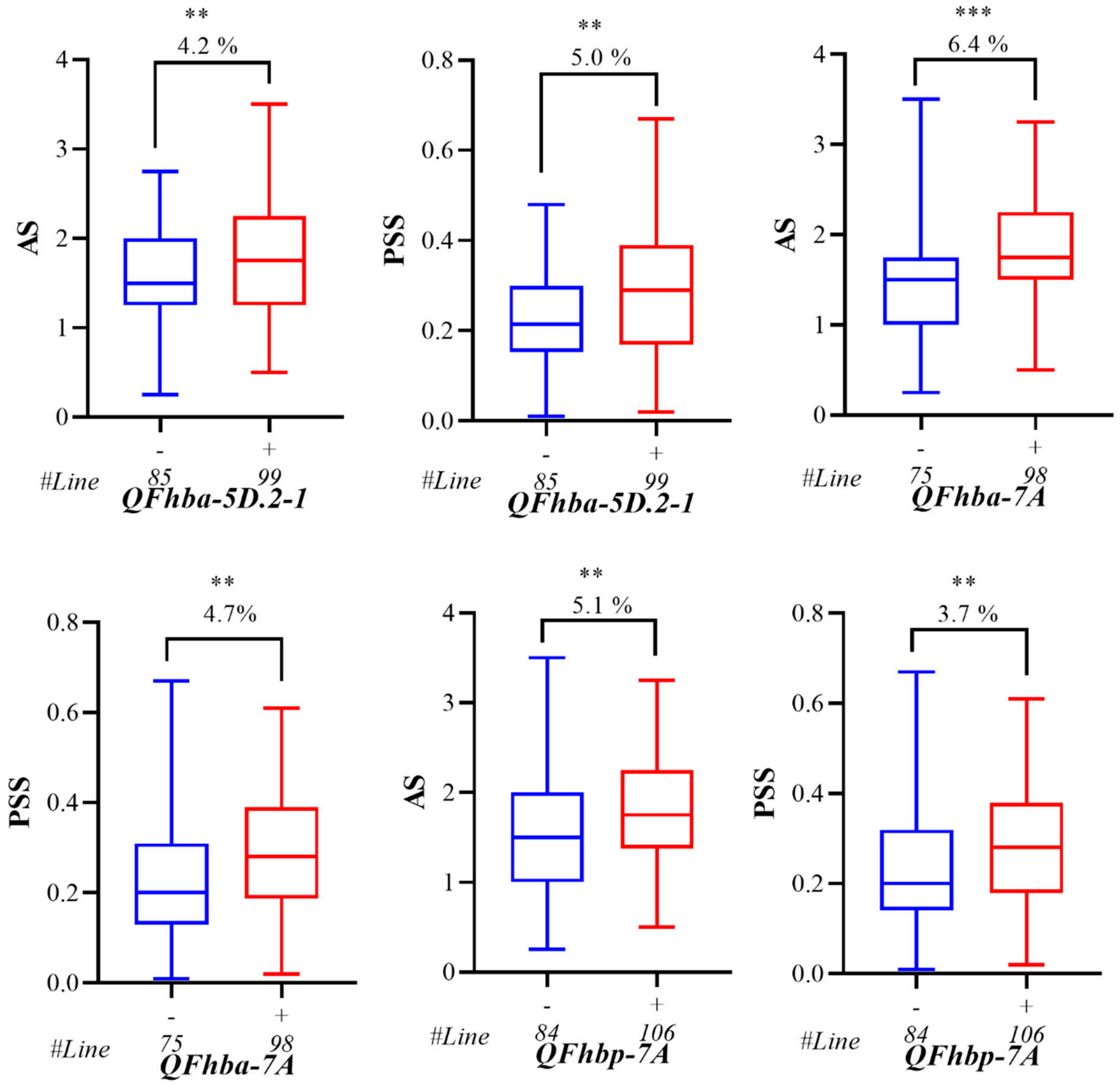

Figure S3. +: Resistance allele of the corresponding flanking marker derived from XN1376; -: susceptible allele of the corresponding flanking marker derived from XY81; *** $p<0.0001$, ** $p<0.001$, * $p<0.05$; AS, average severity; PSS, percentage of symptomatic spikelets.
